# Editorial: Advances in Farm Animal Genomic Resources

**DOI:** 10.3389/fgene.2015.00333

**Published:** 2015-11-24

**Authors:** Stéphane Joost, Michael W. Bruford, The Genomic-Resources Consortium

**Affiliations:** ^1^Laboratory of Geographic Information Systems, School of Architecture, Civil and Environmental Engineering, École Polytechnique Fédérale de LausanneLausanne, Switzerland; ^2^School of Biosciences, Cardiff UniversityCardiff, UK; ^3^Sustainable Places Research Institute, Cardiff UniversityCardiff, UK

**Keywords:** genomic resources, conservation of genomic diversity, data integration, GIS, next generation sequencing, social sciences, disease resistance, sustainable breeding

Livestock conservation is changing rapidly in light of policy developments, climate change, and diversifying market demands. The last decade has seen a step change in technology and analytical approaches available to define and manage Farm Animal Genetic Resources (FAnGR). However, these rapid changes pose challenges for FAnGR management in terms of technological continuity, analytical capacity, and integrative methodologies. Indeed, high-throughput single-nucleotide polymorphism genotyping is available for all major farm animal species and beyond the technological challenge to deal with these large molecular datasets, their integration with geo-environmental and socio-economic information is key to making sense of the data in practical conditions.

In this context, a 4-year (2010–2014) European Science Foundation (http://www.esf.org) Research Networking Programme “Advances in Farm Animal Genomic Resources” (Genomic-Resources) proposed an action dedicated to the education of young scientists in cutting edge approaches to the characterization, analysis, evaluation, management, and conservation of FAnGR. The RNP funded three summer schools (Italy, Croatia, Austria), three workshops (Switzerland, Iceland, Finland), two conferences (Belgium, United Kingdom), and 26 exchange grants. These actions directly connected a community of 350 researchers to develop activities with the goal to meet two major challenges: (i) training in the use of novel methods able to manage and analyse high-throughput molecular data, and (ii) promoting collaboration between the animal science and social science communities to more efficiently manage FAnGR.

In addition to the activities described above, Genomic-Resources has, in this issue of *Frontiers in Genetics*, fostered scientific contributions applying new methods to genomic and bioinformatics approaches for characterization of FAnGR, enhancing *ex-situ* and *in-situ* FAnGR conservation methods, promoting socio-economic elements of FAnGR conservation, transferring lessons between wildlife and livestock biodiversity conservation and has evaluated the contribution of FAnGR to a transition in agriculture (agro-ecology). The 31 articles can be broadly attributed to six different topic areas.

The first topic area contains general papers dealing with the identification of questions of highest priority for FAnGR research during the coming decade (Bruford et al., 2015), the common management challenges shared by livestock breeds and threatened natural populations (Kristensen et al., 2015), the transformation of FAnGR from economic, ecological, and scientific into political entities (Tamminen, 2015), and on the impact of climate change on genetic resources, which constitute the livelihoods of around 1 billion people worldwide (Boettcher et al., 2015).

The latter paper links to a section of papers dedicated to Africa, a continent where livestock genetic resources are particularly endangered and where climate change poses a major threat. The need to increase short-term productivity is prompting the substitution of local breeds by cosmopolitan ones, with the consequence of breeders becoming dependent on expensive external inputs (Colli et al., 2014) instead of making greater use of the well-adapted livestock already living in Africa (Hanotte et al., 2010). Here Benjelloun et al. (2015) characterize neutral genomic diversity and selection signatures in local Moroccan goat populations illustrating the use of whole genome sequence data. To the South, in Burkina Faso, Trypanosomosis transmitted by tsetse-flies is a cause of productivity reduction in cattle. To better understand resistance to the disease, Smetko et al. (2015) compare levels of zebu and taurine admixture in genomic regions possibly involved in trypanotolerance. Eastwards, in Kenya, Kim and Rothschild (2014) analyze the ancestry of local cattle admixed with imported breeds including Guernsey, Norwegian Red, and Holstein to provide useful information for dairy breeding. In Malawi, Zimbabwe, and South Africa, Khanyile et al. (2015) focus on village chicken production and investigated the genetic structure and diversity in more than 300 individuals with a High-density SNP assay to extract valuable information useful for indigenous animal genetic resources management. Finally, in South Africa Makina et al. (2014) investigate genetic diversity and population structure among six cattle breeds using a whole genome SNP panel to examine the possible valuable distinctiveness of indigenous South African breeds likely to cope with climate change.

To stress the important role of social science and its links with animal science in FAnGR management, Genomic-Resources encouraged interaction between the fields (see http://www.genresandpit.eu/) and solicited contributions to this special issue to illustrate the inputs of these disciplines. Key contributions within this scope constitute part of section 3. Ahmadi et al. (2015) highlight the role of decision support systems—used for FAnGR prioritization for example—to integrate technical and social aspects of farming practices. The social dimension of FAnGR conservation is also exemplified by citizen's willingness to pay for conservation programs according to their preferences for native breeds, as shown by Pouta et al. (2014). Section 3 is complemented by papers dealing with agro-ecology. As argued by Tixier-Boichard et al. (2015), the application of agro-ecology to livestock production requires a change of scale in breed management, and represents a social rather than a genetic challenge. Then, concerned with the physical dimension of agro-ecology, Kantanen et al. (2015) review the main changes in Nordic agro-climatic conditions caused in part by livestock production, stressing the importance of animals' ability to adapt.

The fourth section is dedicated to the analysis of demographic events that have shaped cattle diversity. Utsunomiya et al. (2015) track the milestones of past selection in the bovine genome, Orozco-terWengel et al. (2015) revisit demographic processes in cattle with genome-wide data, and Gargani et al. (2015) show how DNA from archeological remains can be used to interpret the history of ancient populations and their supposed relationship with Chianina and Romagnola, two modern central-Italian breeds. Bosse et al. (2015) investigate different events in the history of the domestication of the Eurasian wild boar (*Sus scrofa*), comparing the genomes of European commercial pigs to their wild ancestors. The section closes with a description of SNeP, a tool to estimate changes in effective population size using genome-wide SNP data which can improve our understanding of population demography in the recent past (Barbato et al., 2015).

The next section in the special issue focuses on local breeds. It is introduced by two mini-review papers on the relevance of genetic improvement for these breeds (Biscarini et al., 2015), and on the opportunity represented by locally-adapted livestock breeds in the United Kingdom as valuable reservoirs of adaptive fitness to face productivity issues under changing climate (Bowles, 2015). Also highlighting the characteristics of local breeds, Broxham et al. (2015) describe the BushaLive project targeting the autochthonous Buša cattle of the Balkans. The use of genomic analysis to manage small endangered populations (Mészáros et al., 2015), the determination of comb color in two Swedish local chicken breeds (Johansson and Nelson, 2015), the genetic characterization of the Terceira Pony from the Azores (Lopes et al., 2015), and resistance to gastrointestinal nematodes in the Iranian Ghezel sheep (Valilou et al., 2015) comprise the other research papers presented in this section.

The final section combines research on tools and approaches used in the context of (industrial) breeding programs. Gutiérrez-Gil et al. (2015) compile a review of studies with more than 1000 selection signatures in mainly beef and dairy breeds, and propose a characterization of these selective sweeps. The other contributions illustrate different applications related to estimated breeding value (Rodríguez-Ramilo et al., 2015), genomic selection (Do et al., 2014; Fragomeni Bde et al., 2014), and genetic differentiation (García-Ruiz et al., 2015).

This research topic has provided a valuable set of papers taking stock of the current advances in farm animal genomic resources worldwide. However, it inevitably lacks contributions in some areas, such as incorporation of Geographic Information Systems (GIS) to integrate complementary data on population genetics, animal husbandry practices, socio-economic and environmental characteristics (Joost et al., 2010) needed to enable the “landscape approach” advocated (Boettcher et al., 2015), and featuring in several of the top 20 questions in farm animal genomics research (Bruford et al. 2015, Table 1, questions #4, #12, #13 and #14). While the integrative function they provide is likely to identify potentially valuable genetic material (Hanotte et al., 2010), GIS and related approaches remain underexploited in FAnGR management.

## Author contribution

SJ initiated the research topic and chaired the corresponding European Science Foundation project, SJ and MB wrote and revised the manuscript, members of the Genomic-Resources consortium managed the ESF project and participated in the editorial process of this research topic.

### Conflict of interest statement

The authors declare that the research was conducted in the absence of any commercial or financial relationships that could be construed as a potential conflict of interest.
